# Evaluating the impact of milk sample-based pregnancy test procedures on milk production and daily time budgets of dairy cows

**DOI:** 10.3168/jdsc.2025-0815

**Published:** 2025-07-30

**Authors:** Sushil Paudyal, Juan M. Piñeiro, Artur Grando Pilati, Douglas Dauscheck, Bhuwan Shrestha, Rajesh Neupane

**Affiliations:** 1 Department of Animal Science, Texas A&M University, College Station, TX 77843; 2 Texas A&M AgriLife Research and Extension Center, Amarillo, TX 79106

## Abstract

Prolonged restraint of dairy cows during pregnancy diagnosis in headlocks can elicit stress responses, potentially compromising animal welfare and productivity. Sample-based pregnancy testing (SBPT) utilizing milk samples obtained at the milking parlor eliminates the necessity for prolonged headlock restraint. The objective of this study was to evaluate the effects of implementing SBPT procedures in commercial dairy farms by assessing milk production and daily time budgets of cows compared with traditional pregnancy testing methods. An experiment was conducted at a USDA-certified organic dairy farm using a repeated crossover design with treatment (TRT: SBPT) and control (CON: transrectal ultrasound) groups in 3 pens over 6 periods. Cows in the TRT group were subjected to IDEXX milk SBPT in the milking parlor, whereas those in the CON group underwent transrectal ultrasound in headlocks for pregnancy diagnosis by a trained veterinarian. Daily milk yield from individual cows was recorded by inline milk meters, and lying behaviors and step counts were recorded using leg-based accelerometers. On the day of the pregnancy test, TRT cows exhibited a 0.45 kg higher milk yield compared with CON cows. Milk yield increased by 1.13 kg on the day after testing and by 0.53 kg per day weekly average in TRT cows. The TRT cows displayed higher daily step counts and lying bout frequencies, but shorter lying bout durations on testing days, although total lying time did not differ significantly between groups. These results suggest that SBPT procedures may facilitate reduced restraint time, leading to improved milk production and altered daily time budgets, thereby potentially reducing prolonged headlock-induced stress for a short term.

## Highlights

• Milk sample-based pregnancy testing procedures increased daily milk production.
• Milk sample-based pregnancy testing procedures increased daily step count and lying bout frequencies.
• Milk sample-based pregnancy testing procedures decreased daily lying bout duration.

## Graphical Abstract

**Figure:**
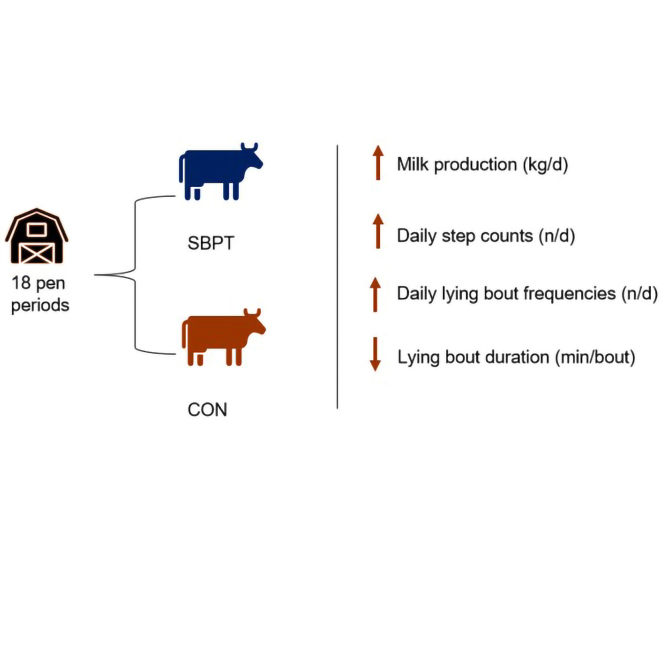
**Summary:** Milk sample-based pregnancy testing procedures (SBPT) increased daily milk yield and cow activity, as evidenced by greater step counts and more frequent lying bouts. These procedures also reduced lying bout duration, suggesting altered rest behavior. Findings support the utility of milk-based testing procedures in enhancing productivity and cow welfare by reducing cow restraint time. CON = control.

Milk sample-based pregnancy tests (**SBPT**) in cattle have been getting attention recently due to their ability in identification of pregnant animals and the reduced need for on-farm experts (Lawson et al., 2014). The milk-based SBPT conducted at the milking parlor further minimizes the need for cows to be restrained in headlocks for pregnancy diagnosis. Cow restraint at freestall headlocks is commonly used during day-to-day management activities. These self-locking stanchions in barns that use fence-line feeding were developed in 1970s (Bolinger et al., 1997) and are extensively used for pregnancy diagnosis, artificial insemination, tail head chalking for breeding, and various veterinary procedures (Arave et al., 1996; Kasimanickam et al., 2018). A single person can effectively control a group of cows by utilizing headlocks, thereby improving labor efficiency and promoting individual animal assessment and care. Although the practice of restraining cows using headlocks is widespread in dairy farms, the restraint is prolonged during pregnancy detection at headgates and the duration varies between herds depending on the expertise and efficiency of personnel for breeding and pregnancy detection purposes, varying between 2 to 4 h in US dairy farms (Papinchak et al., 2022). The specific duration of time an individual cow will be restrained in the headlock also varies by pen within a farm and can be influenced by factors including pen size, handler skills, milking order, or the cows' relative location at the feed bunk after milking. The negative effects of the prolonged head lock-up practices on cow welfare have been previously assessed using indicators that include milk production (Bolinger et al., 1997; Paudyal et al., 2023), reproduction rates (Kasimanickam et al., 2018), disease prevalence, heat stress, lameness, and overall cow behavior (Bolinger et al., 1997; Papinchak et al., 2022). A recent study (Paudyal et al., 2023) identified a 4% increase in milk production in one monthly milk test at a dairy farm when producers ceased the use of headlock restraints. Cows have demonstrated adverse behavioral responses to headlock restraint including repeated escape attempts (Kasimanickam et al., 2018), lying attempts, and vocalizations (Bolinger et al., 1997). Moreover, cows have altered daily lying, eating, and rumination behavior in response to extended head lock-up and rebound effects postrestraint on feeding, drinking, grooming, and aggression behavior (Bolinger et al., 1997). In this study, we evaluate SBPT as a strategy for minimizing prolonged head lock restraints and evaluate its effect on cow performance and cow time budgets.

Early and accurate identification of pregnancy or associated pregnancy loss helps to improve the reproduction efficiency in the dairy herd. In the majority of dairy farms, pregnancy is confirmed by a trained veterinarian either using the transrectal palpation technique or ultrasound examination around 40 to 50 d after insemination (Lavon et al., 2022). This procedure requires a skilled veterinarian, and cows need to be restrained in headlocks. In addition, this process reduces opportunities for cow lying down and resting, which affects cow welfare (Lavon et al., 2022; Papinchak et al., 2022). Additionally, repeated palpation over the long term has been shown to negatively affect veterinarians' well-being, contributing to health issues such as musculoskeletal discomfort and pain in the neck, back, and shoulders (Zeng et al., 2018; Cattell, 2000). Additionally, this procedure currently takes up most of the dairy veterinarian's time, which could be used in herd health monitoring.

Pregnancy loss is an important issue in dairy cows, and detecting it earlier allows for faster reinsemination, ultimately decreasing days open (Santos et al., 2004). The blood sample-based pregnancy test using PAG allows the examination of pregnancy around 28 d after insemination (Green et al., 2005; Wallace et al., 2015). The PAG molecule is produced by placental cells and enters the maternal circulation as early as d 22 to 24 postinsemination and can be detected in plasma, serum, or milk (Lawson et al., 2014; Fricke et al., 2015). The PAG concentration in blood and milk is believed to be an indicator of placental function and predictive of pregnancy maintenance (Doyle et al., 2025). The milk PAG-based pregnancy test at the milking parlor eliminates the need to lock up cows for pregnancy detection (Mayo et al., 2016). In turn, this prevents the discomfort and stress of cows during the blood sample collection.

We hypothesized that decreasing headgate lock-up time by using the milk PAG-based test at the milking parlor would benefit milk yield, lying time, steps, and lying bouts. Consequently, our objectives were to evaluate the effects of minimizing prolonged headlock up restraint using SBPT procedures on milk yield, lying time, steps, lying bouts, lying bout duration in cows tested for pregnancy through milk sample-based tests compared with gold standard method of transrectal ultrasound scanning of contents of the uterus.

All procedures related to animal use were conducted with the approval of the West Texas A&M University Animal Care and Use Committee (IACUC no. #2023.06.001). The experiment was conducted at a USDA-certified commercial organic dairy farm in North Texas between June and July 2023. The farm housed 3,500 Holstein dairy cows in an open dry-lot system, milking 2 times a day with no access to pasture during the study period. A sample size of 18 pen periods was calculated to detect an effect size of 1.5 kg of milk per cow per day (Lavon et al., 2022), assuming a standard deviation of the treatment difference of 1.1 kg, with 80% power and a 5% significance level. The study enrolled 3 open dry-lot pens (average of 250 cows per pen) in a repeated crossover design, with treatment (**TRT**) and control (**CON**) groups alternating across 6 experimental periods. This design yielded a total of 18 pen periods (9 TRT and 9 CON). The experiment was structured to ensure uniformity within sequences, meaning each treatment appeared an equal number of times within each sequence (Table 1). Each pen period was considered the experimental unit for this study. Cows in the TRT groups were tested for pregnancy using IDEXX milk SBPT procedures with milk samples obtained at the milking parlor, subsequently submitted to the IDEXX laboratory (Knoxville, TN) with overnight delivery. From each cow, a 10-mL milk sample was collected after the fore-strip step of the milking process. A total of 39, 8, 39, 18, 38, and 21 milk samples were obtained in each period during the study and submitted overnight for quantitative milk ELISA (IDEXX Laboratories Inc., Westbrook, ME). Cows in the control group were tested for pregnancy using transrectal ultrasound at feed bunk headlocks by a trained veterinarian between d 35 to 42 postinsemination. A leg-based accelerometer (Ice Cube, Ice Robotics Inc., Ireland, UK) that recorded daily cow-level activity behavior was placed on the rear leg of a subgroup of cows (n = 163). Daily lying time, daily step counts, daily lying bouts, and daily lying bout durations were downloaded from the sensors on a weekly basis. Individual milk recording meters at the milking parlor (GEA) were used to obtain daily milk yield per cow for all cows in the study pens. Milking parlor efficiency and parlor turnover time were obtained by timing the milking process on the testing days.

**Table 1:** Schematic representation of treatment (TRT; group that was exposed to the milk sample-based pregnancy test procedures) and control (CON; group that was exposed to the transrectal ultrasound-based pregnancy diagnosis procedures) group distribution under the repeated crossover design used in this study^1^

Pen		Period 1		Period 2		Period 3		Period 4		Period 5		Period 6
A		CON		TRT		TRT		CON		TRT		CON
B		TRT		CON		TRT		CON		TRT		CON
C		TRT		CON		CON		TRT		CON		TRT

1 This design yielded a total of 18 pen periods (9 TRT and 9 CON) spread across the 6 periods.

Data were organized in spreadsheets (Excel 2021, Microsoft Corp., Redmond, WA) and all subsequent statistical analyses were performed using the MIXED procedure in SAS (version 9.4, SAS Institute Inc., Cary, NC) following suggestions for analysis of crossover designs by St-Pierre (2007). The model included milk yield, lying time, step counts, and lying bouts as outcome variables and replications, crossovers, treatments, lactation, and DIM as the independent variables. Cows within the period and sequence were considered as random effects within the model. Interaction effects of treatment by sequence, treatment by period, and pen by period were tested but not included in the final model due to lack of statistical significance. The effect of treatment on daily observations within a period was assessed using repeated measures analysis (REPEATED statement for PROC MIXED) considering an autoregressive covariance structure. Statistical significance was determined at *P* < 0.05.

Overall, cows in the CON groups were restrained in headlocks for an average of 1.7 h, whereas the TRT group was restrained for around 0.6 h on the day of pregnancy detection. The restraint time in the TRT cows was mainly for cow counting, breeding, and tail spray painting. The average parlor turnaround time in the TRT group was around 13.5 min (range 12–14) compared with 11.5 min (range 11–12) in the CON group. This indicates minimal impact of milk sampling procedures on the milking parlor turnaround times.

On the day of the test, cows in the TRT group had 0.45 kg more milk yield than the CON group (*P* = 0.04; Table 2). On the day after the treatment, the daily milk yield was 1.13 kg per cow more for TRT cows than CON cows (*P* < 0.01). The average daily milk yield for the week was 0.53 kg more for the TRT group (*P* < 0.05), indicating an overall positive effect of SBPT on milk production. On the day of testing, cows in the TRT group had a higher daily step count (mean ± SEM; 1,538 ± 129 vs. 1,340 ± 150, *P* = 0.02), as well as a higher number of bouts per day (7.69 ± 0.44 vs. 5.47 ± 0.44, *P* < 0.001). In contrast, the average lying bout duration per day was greater in the CON cows than in TRT cows (mean ± SEM; 55.4 ± 3.32 vs. 63.0 ± 3.35 min, *P* = 0.03) on the test day. However, the daily total lying time of cows was not detected to be different, with lying times of (mean ± SEM) 528 ± 16 and 547 ± 21 min daily for TRT and CON groups (*P* = 0.8), respectively. Within a period, the effect on these variables is predominantly detected immediately following the treatment, with effects diminishing shortly thereafter (see Figure 1).

**Table 2:** Least squares means and SEM for milk production and cow comfort variables in the treatment group that was exposed to a milk sample-based pregnancy test (SBPT) procedure at the milking parlor and the control group that was exposed to transrectal ultrasound-based pregnancy diagnosis at headlocks in an organic dairy farm setting

Item	SBPT	Control	SEM	Treatment *P*-value	Sequence *P*-value	Period *P*-value
Milk yield on d 0 (kg/d)	23.01	22.56	0.21	0.04	0.04	0.80
Milk yield on d 1 (kg/d)	24.69	23.56	0.35	<0.001	0.88	0.93
Average daily milk yield for the week (kg/d)	22.59	22.06	0.23	<0.01	<0.01	0.11
Daily lying time (min/d)	528	547	21	0.85	0.08	0.002
Daily steps (n/d)	1,538	1,340	16	0.05	0.45	0.20
Daily lying bouts (n/d)	7.69	5.47	0.44	<0.001	0.31	0.59
Average lying bout duration (min)	55.4	63.0	3.34	0.03	0.03	0.70

**Figure 1:**
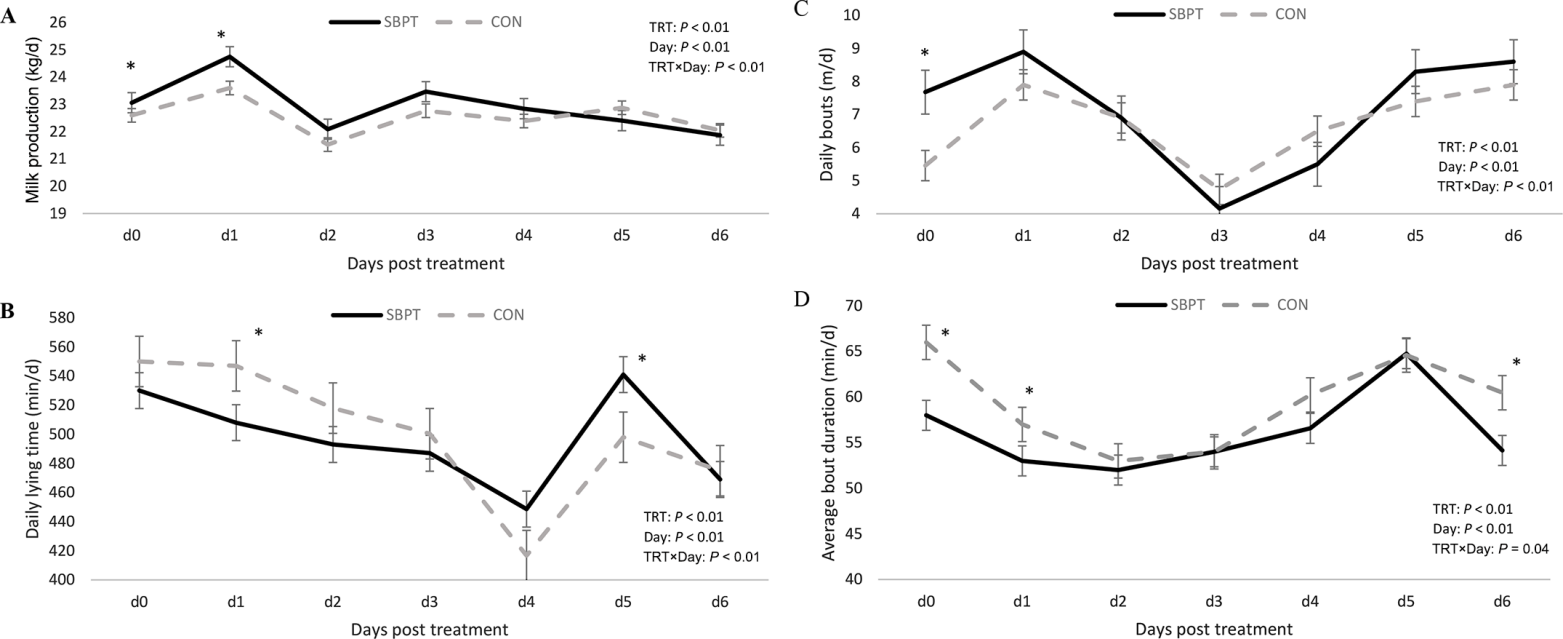
Representation of (A) milk yield, (B) daily lying time, (C) daily bouts, and (D) average bout duration in cows evaluated with milk sample-based pregnancy test (SBPT) procedures in the milking parlor and transrectal ultrasound at headgates (CON) for 6 d after treatment (d 0–6). The SBPT cows displayed higher lying bout frequencies, but shorter lying bout durations on testing days, although total lying time did not differ significantly between groups. Values are presented as LSM ± SEM. Within a day, pairwise comparisons that were significantly different (*P* < 0.05) based on LSD are represented by an asterisk.

The results from this study demonstrate the potential benefits of implementing SBPT procedures in dairy farms. The observed increase in milk production on the day after pregnancy testing and the higher weekly average milk yield in the SBPT group that did not undergo head lock-up compared with the control group are consistent with previous findings by Rulquin and Caudal (1992). The study by Paudyal et al. (2023) also reported increased milk yield in cows without head lock on freestall barn as compared with cows with head lock for 2 h/d, which suggests that removing head lock-up strain improves milk production. A recent study by Lavon et al. (2022) also detected a numerically greater milk production as high as 1.5 kg when using SBPT, although they failed to detect a statistical significance of this difference. Similarly, Bolinger et al. (1997) also indicated no difference in milk production between cows with head lock in self-locking stanchions and without headlock. We attribute the overall lower total milk production per cow reported in this study to the organic milk production system, in addition to the seasonal effect of heat during the data collection period (June–July months). Therefore, the improvement achieved in milk production should be interpreted within the management conditions of this study. Further, the smaller difference in the milk yield on the day of head lock-up could be attributed to the effect captured for one milking (evening milking) compared with both milkings on subsequent days.

The lower daily step counts and reduced lying bout frequency observed in the cows exposed to longer headlock group may be suggestive of physical impediment, lethargy, and reluctance to activity in these cows, as restrained cows have been shown to exhibit behavioral changes such as fewer escape attempts and vocalizations (Bolinger et al., 1997; Kasimanickam et al., 2018). The step count was inconsistent with the available literature, in the study by Lavon et al. (2022), that the number of steps per hour between 2 consecutive milking was greater in rectal palpation as compared with SBPT; however, our study found greater daily steps in the SBPT groups than rectal palpation groups. This may be due to evaluation at the granularity per hour compared with daily values used in our study, which can mask the hourly differences. Lavon et al. (2022) also observed the difference in average lying time between 2 consecutive milkings to be nonsignificant in the milk-based test group and rectal palpation group, which is consistent with our findings. Furthermore, the lying bouts are greater in the TRT group as compared with CON group, which is similar to the reported results from Lavon et al. (2022), where they observed a greater lying number in the SBPT group.

Compared with the CON group, the observations that TRT cows had greater steps, lying bouts, and shorter lying bouts duration suggests that these cows had more opportunity to express their behaviors likely due to the decreased restraint time (Vasseur et al., 2012). Conversely, CON cows seem to have compensated for the increased restraint time and decreased lying bouts by increasing lying bout duration. Previous studies have demonstrated that lying behavior is a crucial component of cow comfort and that prolonged standing times can lead to increased risk of lameness and other health issues (Douphrate et al., 2013). Although the prolonged head lock-up events due to pregnancy diagnosis occur weekly and we demonstrated the effect to be short-lived, it is important to consider the potential long-term implications of these altered lying patterns on cow welfare and productivity.

It is important to note that the study was conducted in an open dry-lot system at a USDA-certified organic dairy farm, which may have contributed to some of the observed effects on milk production and cow behavior. Thus, as the production system and environment may have effect on the results, some of these observations should be interpreted within the context of this study. Further research into different housing systems and management practices would be valuable to validate the applicability of SBPT procedures across a wider range of dairy operations. Additionally, potential economic benefits of implementing SBPT, such as reduced labor costs and veterinary expenses, could provide further incentives for adoption by dairy producers (Gwazdauskas, 2002; Manríquez et al., 2022). However, a comprehensive economic evaluation should include the costs as well as the benefits of both these systems when considering accuracy at the given moment. Although milk yield is something relevant for dairy farmers, this “transient” change in milk yield could have little effect on annual profit, as the pregnancy testing frequency influences the potential milk losses. Further extensive evaluation to include milk components (milk fat and milk protein) as well as somatic cell counts should be conducted, as these factors highly influence the milk pricing in the US dairy systems.

Transrectal ultrasound scanning of the contents of the uterus is a well-established method of pregnancy diagnosis and is considered the gold standard when compared with other methods (Roberts et al., 2015). Pregnancy detection using serum- and milk-based PAG has different levels of accuracy (Lawson et al., 2014) because PAG concentrations in maternal circulation initially increase until approximately d 36, then decline until d 60, and increase again thereafter to reach maximum concentration before parturition (Green et al., 2005; Pohler et al., 2013; Ricci et al., 2015). This is further affected by factors including parity of cows, milk production, stage of gestation (Ricci et al., 2015), and dam genetic merit (Doyle et al., 2025). Therefore, consideration of the timing of pregnancy evaluation is crucial when making conclusions on the benefits of these systems.

The crossover design in this study ensures that the confounding effects of pen and cows in the treatment groups are minimized, by having all pens sequentially exposed to both treatment and control conditions. The main disadvantage of a crossover design is the opportunity for residual carryover effects. However, the withdrawal period of 6 d used in this study likely minimized the carryover effect. The impact of the restraint for a day in a week will not last more than 6 d to have an influence as a carryover effect on the treatment groups (Munksgaard et al., 2005; Cooper et al., 2007). Further, this study design is strongly balanced to first-order carryover effects because each treatment precedes every other treatment the same number of times, increasing the robustness of the results.

A comprehensive cost-benefit analysis is beyond the scope of this short communication article, and should include the costs of milk tests and palpation, sensitivity and specificity of the tests, benefits of milk production, timeliness of the results, early detection of pregnancy, and long-term reproductive impacts. A simplified economic assessment based on the variables measured in this study estimates that an additional 12,958 kg of milk (0.53 kg/d × 7 d × 3,500 cows) could be produced, resulting in approximately $8,015 in added revenue based on the 2023 organic milk price of $28/hundredweight. This estimate should be carefully interpreted in the context of broader economic evaluation, income over feed costs, and the costs of pregnancy diagnosis, approximately $2 to $5 per cow for rectal palpation and $3 to $5 per cow for the Alertys milk-based testing system depending on factors including farm location and number of tests.

We conclude that SBPT procedures facilitate reducing the restraint time for cows managed in headlocks, which could be associated with increased milk yield and behavioral metrics, as indicated by the increased daily lying bouts and steps.
